# Supplementary material for: A Single Shot Pre-fusion-Stabilized Bovine RSV F Vaccine is Safe and Effective in Newborn Calves with Maternally Derived Antibodies
Source: Vaccines (Basel). 2020 May 18;8(2):231. doi: 10.3390/vaccines8020231 (PMC7349975; doi:10.3390/vaccines8020231)
Supplement: Supplementary file 1 [file vaccines-08-00231-s001.pdf]

Table S1. List of calves and allocation to experimental groups based on BRSV-MDA titers and age.

Group	ID	Age of the Calves at Time of vaccination (Days)	IgG in Sera (g/dL) [1]	MDA [2]	Systemic Reaction to Vaccination Δ(TD1-TD0) [3]	Local Reaction to Vaccine [4]
ISA61	6431	23	6.3	6.2	2.1°C	none
	6400	50	6.3	15.2	0.2°C	none
	6417	40	6.8	33.9	0.5°C	none
	6567	34	6.8	74.3	0°C	none
	6434	22	6.8	56.9	1.9°C	none
	6403[#]	48	5.8	44.6	0.0°C	none
preF/ISA61	6422	35	5.8	2.1	0.9°C	none
	6405	48	6.8	13	0.0°C	none
	6568	38	6.3	31	1.1°C	none
	6407	46	6.8	74.5	0.4°C	none
	6427	27	7.9	57.8	0.1°C	none
	6559	24	5.8	65.7	1.5°C	none
PreF+N/ISA61	6410	44	5.8	2.1	0.9°C	none
	6562	26	6.8	19.5	0.7°C	none
	6425	30	6.3	39.9	1.6°C	none
	6401	50	6.8	67.3	1.2°C	none
	6570	40	7.4	57.3	1.0°C	none
	6561	26	7.4	56	1.2°C	none

[1] The refractive index of calf sera (collected 48 h after birth) was measured with a refractometer and converted to IgG concentration (g/dL). [2] BRSV-specific MDA were measured in sera collected 2 weeks before vaccination and expressed as % COD of the positive BRSV serum provided with the Svanova commercial kit. [3] The rectal temperature of calves was measured the day before vaccination, the day of vaccination (D0, 2016/02/16) and during 3 days after vaccination. The biggest increase was detected between D0 and D1. [4] The local reaction to vaccine was also recorded daily during 5 days (D-1 to D3). [#] calf 6403 died 3 weeks after vaccination (unknown cause, enterotoxemia suspected).

Table S2. Levels of BRSV RNA in nasal secretions and BAL.

Group	ID	BRSV RNA	
		AUC Nasal Secretions (D0-D7) [1]	BAL [2]
ISA61	6431	6194	163325
	6400	11620	313226
	6417	3109	518461
	6567	678	196658
	6434	25	278787
preF/ISA61	6422	2	0
	6405	0	0
	6568	58	0
	6407	425	0
	6427	0	0
	6559	0	0
PreF+N/ISA61	6410	0	0
	6562	3822	6918
	6425	0	0
	6401	0	0

6570	44	5
6561	221	79

[1] BRSV RNA titers expressed as $TCID_{50}$ equivalents/mL (eq) in nasal secretions determined by the area under the curve from day 0 to day 7 post BRSV challenge. [2] RNA titers expressed as $TCID_{50}$ equivalents/mL (eq) in BAL. The cut off of the PCR is 0.7 $TCID_{50}$ eq.

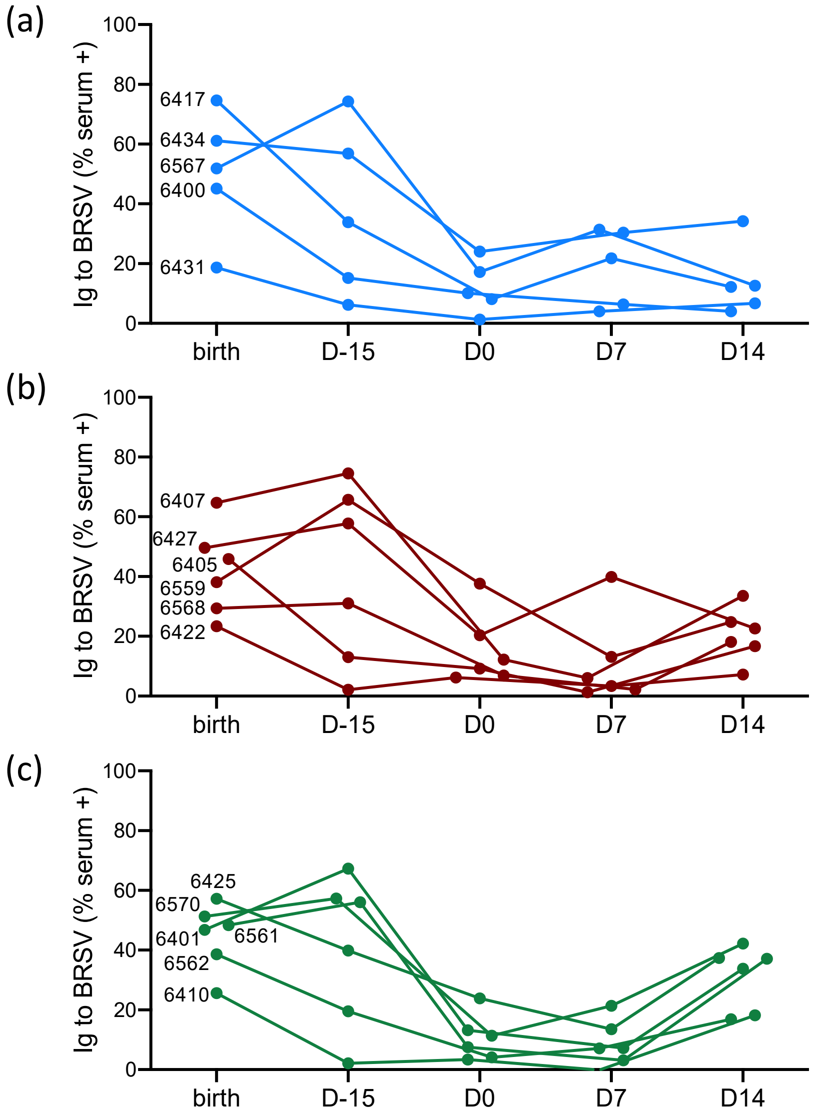

Figure S1. Kinetic of BRSV-specific antibody in calves from birth up to 2 weeks after vaccination.

Calves were vaccinated IM with preF or preF+N in ISA61 adjuvant, or mock (ISA61 alone) (D0). Blood was collected 48h after birth, 2 weeks before vaccination (D-15), at the time of vaccination (D0), 1 and 2 weeks after vaccination (D7 and d14). BRSV-specific Ab titers were expressed as % COD of the positive BRSV serum provided with the Svanova commercial kit. The decline observed from birth up to one week after vaccination can be related to the progressive disappearance of MDA. Calf ID is indicated in front of each plot. (a) Control group (ISA61) (b) preF group (preF formulated with ISA61) (c) preF+N group (preF and N formulated with ISA61)
